# Ecdysteroid-Dependent Expression of the *Tweedle* and *Peroxidase* Genes during Adult Cuticle Formation in the Honey Bee, *Apis mellifera*

**DOI:** 10.1371/journal.pone.0020513

**Published:** 2011-05-31

**Authors:** Michelle P. M. Soares, Fernanda A. Silva-Torres, Moysés Elias-Neto, Francis M. F. Nunes, Zilá L. P. Simões, Márcia M. G. Bitondi

**Affiliations:** Departamento de Biologia, Faculdade de Filosofia, Ciências e Letras de Ribeirão Preto, Universidade de São Paulo, Ribeirão Preto, São Paulo, Brazil; Instituto Nacional de Câncer, Brazil

## Abstract

Cuticle renewal is a complex biological process that depends on the cross talk between hormone levels and gene expression. This study characterized the expression of two genes encoding cuticle proteins sharing the four conserved amino acid blocks of the Tweedle family, *AmelTwdl1* and *AmelTwdl2*, and a gene encoding a cuticle peroxidase containing the Animal haem peroxidase domain, *Ampxd*, in the honey bee. Gene sequencing and annotation validated the formerly predicted *tweedle* genes, and revealed a novel gene, *Ampxd*, in the honey bee genome. Expression of these genes was studied in the context of the ecdysteroid-coordinated pupal-to-adult molt, and in different tissues. Higher transcript levels were detected in the integument after the ecdysteroid peak that induces apolysis, coinciding with the synthesis and deposition of the adult exoskeleton and its early differentiation. The effect of this hormone was confirmed *in vivo* by tying a ligature between the thorax and abdomen of early pupae to prevent the abdominal integument from coming in contact with ecdysteroids released from the prothoracic gland. This procedure impaired the natural increase in transcript levels in the abdominal integument. Both *tweedle* genes were expressed at higher levels in the empty gut than in the thoracic integument and trachea of pharate adults. In contrast, *Ampxd* transcripts were found in higher levels in the thoracic integument and trachea than in the gut. Together, the data strongly suggest that these three genes play roles in ecdysteroid-dependent exoskeleton construction and differentiation and also point to a possible role for the two *tweedle* genes in the formation of the cuticle (peritrophic membrane) that internally lines the gut.

## Introduction

Insects grow and differentiate from the larval to the adult stage through periodic degradation of the exoskeleton (or cuticle) and replacement by a new one. Each episode of cuticle renewal, or molt, comprises a series of events mainly marked by the detachment of the cuticle from the subjacent epidermis (apolysis), the synthesis and secretion of the components of the new cuticle by the epidermal cells, and the ecdysis or shedding of the old cuticle [1]. This cyclic re-construction of the cuticle is a complex task involving the expression of genes for extensive synthesis of structural cuticle proteins (CPs) and enzymes with roles in cuticle pigmentation (darkening) and sclerotization (hardening). The pigmentation and sclerotization pathways involve the activity of enzymes including tyrosine hydroxylase [2] and dopa-decarboxylase [3], [4], [5], which participate in the biosynthesis of catecholic sclerotization precursors that are oxidized to quinones by the action of phenoloxidases [6], [7], [8], laccases [9], [10], [11], [12] and peroxidases [13]. Quinones are the precursors of melanin in the process of cuticle pigmentation, and they also interact to form covalent bonds with amino acid residues of CPs in cross-linking reactions for sclerotization and stabilization of the cuticle [14], [15], [16].

The coordination of cuticle renewal is known to be controlled by the ecdysteroid hormone 20-hydroxyecdysone [17], [18], [19]; its precursor, ecdysone, and other structurally related compounds are only active as molting hormones in a few organisms [20]. The study of the molecular mechanism of ecdysteroid action in *Drosophila melanogaster* demonstrated that a pulse of ecdysteroids mediated by the heterodimeric complex formed by the ecdysone receptor (EcR) and ultraspiracle (Usp) triggers expression of the so-called “early” genes for production of regulatory proteins, which in turn activate the “late” or effector genes in a secondary response [21], [22]. Among other presumed effector genes or late-response genes are those encoding many CPs and cuticular enzymes [23]. Data from *Manduca sexta* have been used to construct a proposed neuroendocrine model for the regulation of molting events that may apply to insects in general. According to this model, the increase in ecdysteroid levels that induces apolysis and early gene expression concomitantly makes the insect central nervous system (CNS) sensitive to the pre-ecdysis hormone and ecdysis-triggering hormone produced by the tracheal Inka cells. The release of both hormones and the release of several CNS neuropeptides, including the eclosion hormone (involved in ecdysis), are induced by late gene expression, which occurs when ecdysteroid titers decrease to baseline levels [24]. Another neuropeptide, bursicon, is critical for the late events of cuticle differentiation and causes cuticle expansion and tanning [25], [26], [27]. Therefore, molting initiation is ensured by the increase in ecdysteroid titer, but the synthesis of a new cuticle and ecdysis only take place when the titer of ecdysteroids decreases after the peak [28], [29], [30], [31]. This sequence of events triggered and timed by ecdysteroids, which includes many other molecular components in addition to those mentioned above, underlies the process of cuticle renewal.

Whole genome sequencing of insect species has allowed prediction of genes potentially involved in cuticle structuring. Predicted CP sequences were included in the cuticle proteome database [32] (http:/bioinformatics2.biol.uoa.gr/cuticleDB/index.jsp), and a recent review by Willis [33] summarized the current state of knowledge about CPs in arthropods. Overall, CPs have mainly been predicted from assembled insect genomes and from the conceptual translation of cDNAs and ESTs. CPs were designated as cuticle components based on their similarity to the so-called authentic CPs, i.e., those identified by biochemical methods in cuticle extracts. The classification of CPs into twelve structurally distinct protein families demonstrates their diversity within and amongst species.

Among the insect species with sequenced genomes, the honey bee, *Apis mellifera*, has the lowest number of genes potentially encoding the major and best-known class of CPs, i.e., the CPR proteins carrying the R&R Consensus sequence [34]. Whereas 32 CPR genes were found in the honey bee genome, 62 of these genes were found in another hymenopteran, the parasitoid wasp *Nasonia vitripennis*, and this number rises to 101, 148 and 156 in *D. melanogaster*, *Bombyx mori* and *Anopheles gambiae*, respectively [33]. The lower diversity of structural CPR proteins in the honey bee has been tentatively explained by the fact that this social insect lives inside a protective hive during most of its life cycle [35].

Although many CP genes have been predicted from insect genomes, most of these genes have not yet been experimentally validated. To our knowledge, only one of the honey bee CPR predicted genes, *AmelCPR14*, has been validated by sequencing of its CDS (coding sequence) [36]. Another cluster of three genes containing sequence motifs characteristic of genes encoding CPs, in addition to other features, was also identified in the honey bee and included in the separate class of *apidermin* cuticle genes [37].

We are particularly interested in studying the molecular basis of formation and differentiation of the adult stage-cuticle. In comparison to the larval and pupal cuticles, the construction of the definitive cuticle in the honey bee (and other insects) is more complex, mainly due to the onset and progress of pigmentation that occurs in concert with an intense process of sclerotization, thus resulting in striking changes in chemical and structural properties [38], [39]. Toward this main goal, we previously characterized the structure and expression of the above-mentioned CPR gene, *AmelCPR14* [36] and a laccase gene, *Amlac2* [11]. These studies established the relationship between the expression of both genes and the ecdysteroid titer that induces apolysis and triggers the biosynthesis and differentiation of the adult cuticle. The current study describes the primary structure, developmental expression and ecdysteroid-dependent regulation of three other genes, two of which belong to the Tweedle CP family, *AmelTwdl1* and *AmelTwdl2*, and the other encoding a cuticular peroxidase, *Ampxd*. Expression of these genes was studied in the thoracic, abdominal and wing integument in the context of the sequential events occurring during the last molting cycle and leading to adult cuticle synthesis and differentiation. In addition, expression of these genes was investigated in the trachea and gut, which are internally lined by cuticle. Characterization of the expression of these genes provided insights into the roles of the Tweedle protein family and a peroxidase in the cuticles making up the exoskeleton and lining internal organs, and it also expanded the list of validated cuticle genes in the honey bee.

## Materials and Methods

### Genes identification and bioinformatic analyses

BLAST searches using as query a CP sequence from *B. mori*, BAE06189.1 [40] allowed identification of the two *tweedle* genes in the honey bee genome. The *peroxidase* gene was identified by using peroxidase protein sequences from *Aedes aegypti* (EAT46477.1) and *Culex quinquefasciatus* (EDS26535.1). TBLASTN searches were performed against the honey bee genome version 4.0 [35] to check chromosomal region (linkage group). BLASTN and BLASTP searches were performed against the *A. mellifera* Official Gene Set database Pre_Release2 to identify predicted sequences. All these information were used to define each gene structure using Artemis 7.0 plataform [41]. Primers were designed along the annotated sequences (File S1) and used for cDNA amplification in semiquantitative (sq) and quantitative (q) RT-PCRs, and also for cDNA sequencing. Sequenced fragments were assembled using Sequencher 3.1 software. Consensus cDNA sequence for each gene was used to refine and validate the annotation. Based on Expasy softwares (http://expasy.org/tools), translation products were deduced from the annotated CDSs and used to compute theoretical molecular mass, isoeletric point, amino acid composition, and also served as input to check potential signal peptide using SignalP 3.0 (http://www.cbs.dtu.dk/services/SignalP/). The deduced proteins and insect orthologs for *tweedle* and *peroxidase* genes were used for further alignment analysis (ClustalW 2 - http://www.ebi.ac.uk/Tools/msa/clustalw2/) and identification of conserved domains.

### *A. mellifera* and tissue dissections

Africanized honey bee workers were collected from colonies maintained at the Experimental Apiary of the University of São Paulo in Ribeirão Preto, SP, Brazil. Pupae and pharate adults were staged according to the criteria established by Michelette and Soares [42], and dissected for separation of the thoracic and abdominal integument (only the dorsal portion), wings, trachea and gut. Total RNA was extracted from samples prepared with 6 thoracic or abdominal integuments, 15 fore wing pairs, and also from samples prepared with trachea or gut dissected from 10 abdomens. Some RNA samples were prepared by pooling three whole bodies of pupae and pharate adults during the successive phases of development.

### Abdominal ligature

Newly ecdysed pupae (Pw phase) were submitted to a ligature between thorax and abdomen. After ligation, they were maintained in Petri dishes lined with filter paper, in an incubator, at 34°C and 80% relative humidity. Development in comparison with non-ligated controls, which were maintained at the same conditions of temperature and humidity, was followed during pupal and pharate adult stages. During this time, ligated and non-ligated bees were periodically taken for dissection of the abdominal integument for total RNA extraction.

### Semiquantitative RT-PCR analysis (sqRT-PCR)

Total RNA was extracted using Trizol (Invitrogen) following manufacturer instructions. The extract was subsequently incubated in the presence of 3 units of RNase-free DNase (Promega) for 40 min at 37°C to eliminate contaminant DNA, and for 15 min at 70°C to inactivate this enzyme. First-strand cDNA was synthesized by reverse transcription using 2.5 µg of total RNA, SuperScript II reverse transcriptase and the oligo (dT_12–18_) primer (Invitrogen). Negative control reactions, without the reverse transcriptase, were also prepared and analyzed in parallel to check for DNA contamination. Aliquots of first-strand cDNAs were used in PCR reactions with PCR Master mix (Promega) and specific primers (File S1). All primer pairs were designed to span at least one intron. Thus, contamination by genomic DNA, if any, could be easily identified after electrophoresis of RT-PCR products by detection of an amplicon size heavier than expected. The thermal cycling program consisted of 2 min at 94°C followed by 30 cycles of 30 s at 94°C, 1 min at 60°C, 1 min at 72°C and a final extension step at 72°C for 10 min. The number of PCR-cycles was previously tested to avoid saturation. The PCR products were analyzed by electrophoresis in 1% agarose gels containing ethidium bromide and visualized using KODAK EDAS 290.

A constitutively expressed *A. mellifera actin* gene, *Amactin* [43] (see File S1 for accession number and primers), was used to control cDNA loading and to correct for differences in cDNA amounts. In this case, the thermal cycling program was as follows: 2 min at 94°C, 25 cycles of 30 s at 94°C, 45 s at 62°C, 1 min at 72°C and a final extension step at 72°C for 10 min.

### Real-time RT-PCR analysis (qRT-PCR)

*AmelTwdl1*, *AmelTwdl2* and *Ampxd* transcripts were quantified in the thoracic integument and in other tissues (trachea and gut) by using a 7500 Real Time PCR System (Applied Biosystems) and the ΔΔC_T_ method by which the relative amount of transcripts is given by 2^-ΔΔC^_T_ (Applied Biosystems User bulletin #2 [44]). The gene encoding a ribosomal protein, *Amrp49*, (see File S1 for accession number and primers), which is expressed in similar levels during honey bee development, and was validated as being suitable for normalizing real time RT-PCR data [43] was used as the endogenous reference. The primers used for the reference and target genes (File S1), were designed to span at least one intron, thereby serving as control for genomic DNA contamination. Previously, validation experiments were performed to verify the efficiencies of amplification of the targets and endogenous reference. Using serial cDNA dilutions, the efficiency (E) of the reactions was calculated (E = 10^(-1/slope)^) for each gene and showed to be approximately equal. Amplification was carried out in a 20 µl reaction volume, containing 10 µl SYBR Green Master Mix 2x (Applied Biosystems), 1 µl cDNA (prepared from total RNA extracted as described for RT-PCR analysis), 7.4 µl water, and 8 pmol of each gene-specific primer. Reactions not including the SuperScript II reverse transcriptase (Invitrogen), or cDNA template, were prepared as negative controls. The PCR conditions were 50°C for 2 min and 95°C for 10 min followed by 40 cycles of 95°C for 15 s, and 60°C for 1 min. Each run was followed by a melting curve analysis to confirm the specificity of amplification and absence of primer dimers. To check reproducibility, each SYBR green assay was done in technical duplicate and repeated with three independent biological samples. The baseline and threshold were correctly set to obtain accurate C_T_ values, which were exported into a MS Excel spreadsheet (Microsoft Inc.) for 2^-ΔΔC^_T_ calculation.

### Cloning and Sequencing

Using specific primers for *AmelTwdl1*, *AmelTwdl2* and *Ampxd* (File S1), cDNA regions were amplified and electrophorized. PCR products were then extracted from the agarose gels, purified with Perfectprep Gel Cleanup kit (Eppendorf), and cloned into pGEM-T Easy Vector (Promega). Insert-containing plasmids were sequenced using M13 forward and reverse universal primers. Dideoxy sequencing was performed in an ABI Prism 310 DNA Analyser using BigDye Terminator v3.0 Cycle Sequencing Ready Reaction (Applied Biosystems).

### SDS-PAGE and Western Blot

The presence of the AmelTwdl1 protein was investigated in the thoracic and abdominal integuments during pupal and pharate adult development. Total protein was extracted by homogenizing integuments from staged bees in 100 µl extraction buffer (Tris-HCl 0,25 M and 2% SDS, pH 6,8). Following incubation on ice during 2 h, the extracts were centrifuged at 15,000× *g*, at 4°C for 5 min and the respective supernatants were collected for total protein quantification using bicinchoninic acid [45]. Aliquots containing 10 µg of total protein were subjected to SDS-PAGE according to Laemmli [46], with some modifications: the 7.5–15% polyacrylamide gradient gels (100×120×0.9 mm) were prepared without SDS, which was added only to the running and sample buffers. Electrophoresis was carried out at a constant current of 15 mA at 7–10°C. Blotting was performed according to the protocol described by Towbin *et al*. [47]. Electrophoretically separated proteins were transferred to nitrocellulose membranes (ImmunBlotTM PVDF Membrane, Bio-Rad), which were subsequently incubated in a 1∶100 diluted primary antibody against the BmGRP2 protein from *B. mori* cuticle. Membrane-immobilized AmelTwdl1 was detected with Horseradish Peroxidase (HRP) labeled secondary antibody diluted 1∶12000 (ECL kit, Amersham Biosciences), and the corresponding fluorescent band was visualized by exposing membranes to a Kodak XR-Omat film.

### Electrophoretic characterization of peroxidase activity in thoracic integument samples

Pools of ten thoracic integuments (only the dorsal portion) dissected from pupae and pharate adults were frozen in liquid nitrogen, pulverized with the aid of a glass pestle, and homogenized in 200 µl extraction buffer (50 mM sodium phosphate pH 6.5, 1% SDS and 4 M urea). After 20 min at room temperature, these samples were centrifuged at 10,000× *g* for 10 min at 4°C. The supernatants were mixed with sample buffer (1.25 ml 0.25 M Tris-HCl pH 6.8, 500 ml saccharose 70%, 0.1 g SDS, 100 ml bromophenol blue, 3 ml water) and used for electrophoresis, performed as described in item 2.6, but using 7.5% polyacrylamide gels. Peroxidase activity was detected in these non-heated samples after incubation of the polyacrylamide gels in 5 mM diaminobenzidine dissolved in 0.2 M sodium acetate buffer pH 6.0 containing 3% H_2_O_2_ [48].

## Results

### Characteristics of two *tweedle* genes and a cuticular *peroxidase* gene in the honey bee

**Figure 1:**
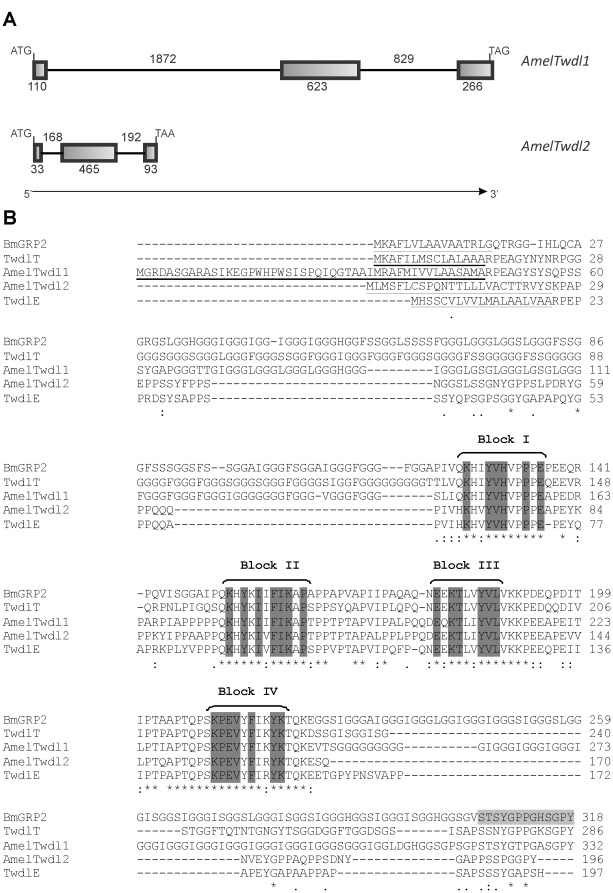
Tweedle: gene structures and alignment of insect tweedle proteins. (A) Schematic representation of the *AmelTwdl1* and *AmelTwdl2* genes. Initiation and termination codons are indicated at the left and right of the figure, respectively. Exons and introns are indicated by boxes and lines, respectively, and the number of nucleotides is shown. The direction of transcription is indicated by an arrow. (B) Alignment (ClustalW 2) of AmelTwdl1 (ACJ38118.1) and AmelTwdl2 (ADK73965.2) amino acid sequences with other Tweedle protein sequences from *B. mori*, BmGRP2 (BAE06189.1), and *D. melanogaster*, TwdlT (AAF56656.1) and TwdlE (AAF52571.2). The four conserved blocks of amino acids are marked in dark gray. The signal peptide region is underlined in all sequences. In light gray is a region of BmGRP2 sequence that was used by Zhong *et al*. (see ref. [40]) to synthesize a peptide for antibody production. This antibody recognized the AmelTwdl1 protein (see Fig. 3 and corresponding text in Results section). Asterisks, colons and dots represent identical amino acid residues, strong- and weak-conservative substitutions, respectively.

Using the *B. mori* BmGRP2 deduced protein (Accession number BAE06189.1, [40]) as a query in BLAST searches, we identified the GB19234 sequence in the genomic region GroupUn.1241. Specific primers were designed for this honey bee sequence, and total RNA was extracted from the thoracic integument. In a reverse transcription procedure, the corresponding cDNA was synthesized, amplified and sequenced. The sequenced cDNA was compared against the corresponding annotated gene *in silico* using the Artemis platform, revealing the exon/intron boundaries (Figure 1A). The CDS spans 999 nucleotides (stop codon included) distributed among 3 exons. Only the last four nucleotides at the 3′ region and the stop codon were not validated by cDNA sequencing. The conceptual primary protein sequence consists of 332 amino acids. It has a molecular mass of 30.81 kDa and a p*I* value of 9.2. The N-terminal signal peptide spans 47 amino acids (see in File S2 the nucleotides validated by sequencing, the deduced amino acid sequence, and the signal peptide region). The deduced protein contains the four conserved blocks of amino acids (Blocks I, II, III and IV) that typically characterize the Tweedle CP family, as observed in the ClustalW alignment with Tweedle proteins from other species of insects shown in Figure 1B. This honey bee gene was then named *AmelTwdl1* (*Amel* was used to conserve the nomenclature given to the *A. mellifera* CPs in the CuticleDB; Twdl is the abbreviation given to *tweedle* genes first described in *Drosophila*), and the GB19234 sequence prediction was validated as a cuticle protein gene. The AmelTwdl1 protein shares 58% similarity (ClustaW score) with BmGRP2 and 60% and 48% similarities with the TwdlT (AAF56656.1) and TwdlE (AAF52571.2) proteins from *D. melanogaster*, respectively [49].

The other honey bee *tweedle* gene, here named *AmelTwdl2*, corresponds to the GB14193 sequence in the genomic region GroupUn.592. This gene was identified by BLASTP analysis using the BmGRP2 and AmelTwdl1 deduced CP sequences as queries. As performed for *AmelTwdl1*, the *AmelTwdl2* cDNA was synthesized using RNA from the thoracic integument, amplified with specific primers based on the GB14193 sequence and sequenced. Computational comparison of the sequenced cDNA against the corresponding annotated gene using the Artemis platform allowed the identification of the CDS formed by 3 exons encompassing 591 nucleotides (stop codon included) (Figure 1A). Only 33 nucleotides at the N-terminus were not validated by CDS sequencing. The conceptual translation product consists of 196 amino acid residues, has a molecular mass of 21.36 kDa and a p*I* value of 8.4, and contains a putative N-terminal signal peptide comprising 25 amino acids (see in File S3 the CDS region confirmed by sequencing, the translated protein sequence, and the signal peptide). AmelTwdl2 contains the four amino acid blocks typical of Tweedle proteins and shares 50% similarity to AmelTwdl1; it also has 41, 42, and 52% similarities to the BmGRP2, TwdlT, and TwdlE proteins, respectively (Figure 1B).

**Figure 2:**
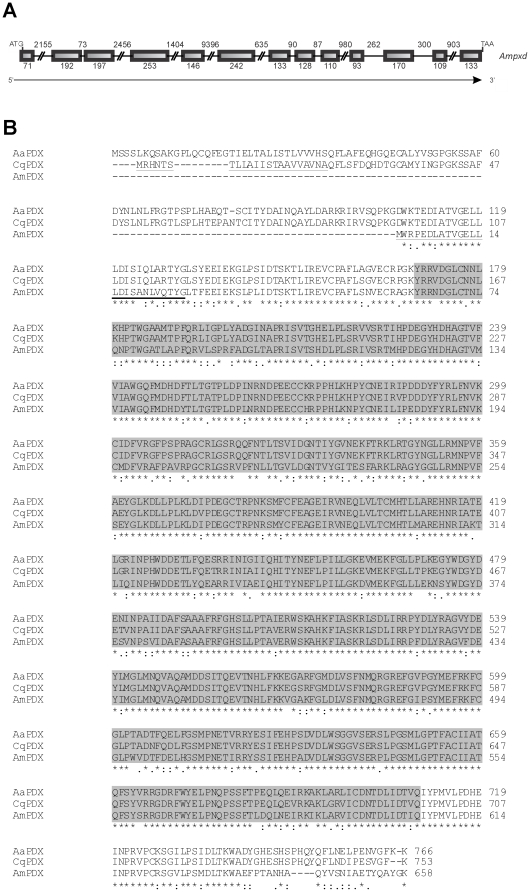
Peroxidase: gene structure and alignment of insect peroxidase proteins. (A) Schematic representation of the *peroxidase* gene from *A. mellifera*. Initiation and termination codons are indicated, as well as exons (boxes) and introns (lines). The number of nucleotides is shown, and the direction of transcription is indicated by an arrow. (B) Alignment (ClustalW 2) of peroxidase sequences from *A. mellifera* (AmPXD, ADE45321.2), *Culex quinquefasciatus* (CqPXD, EDS26535.1) and *Aedes aegypti* (AaPXD, EAT46477.1). The signal peptide region was underlined in the *A. mellifera* and *C. quinquefasciatus* sequences. The region containing the Animal haem peroxidase domain (pfam03098) is marked in grey. Asterisks, colons and dots represent identical amino acid residues, strong- and weak-conservative substitutions, respectively.

BLASTP analysis using the predicted peroxidases of *Aedes aegypti* (EAT46477.1) and *Culex quinquefasciatus* (EDS26535.1) failed to identify a *peroxidase* gene prediction in the Official Gene Set pre_release2 database. However, we verified that in the honey bee genome version 4.0, both dipteran genes aligned to two regions, Group4.3 and GroupUn.1247. To annotate and determine the structure of the honey bee *peroxidase* gene, both linkage groups were reoriented and merged. These two orthologous sequences enabled us to manually predict a gene model. Gene sequencing using primers (primers *Ampxd* f2 and r2, see File S1) flanking regions mapped on both linkage groups, Group4.3 and GroupUn.1247, confirmed that these groups are indeed continuous. The identified *peroxidase* gene spans ∼21 kb of genomic DNA. The annotated CDS has 1977 nucleotides (stop codon included) and is separated into 13 exons (Figure 2A) that potentially encode a protein of 658 amino acids. Expected canonical splice sites (conforming to the GT/AG rule) were found at all exon/intron boundaries. The CDS was partially sequenced, with 1403 of the 1977 nucleotides identified, validating 7 entire and 2 partial annotated exons. These sequence data were submitted to GenBank under accession number GU785071.2 (ADE45321.2 for its conceptual translation product). The Animal haem peroxidase domain (pfam03098) spanning a region of 541 amino acids was identified in the deduced translation product. The predicted N-terminal signal peptide includes 26 residues (see in File S4 the CDS region confirmed by sequencing, the translated protein sequence, and the signal peptide). The molecular mass of the annotated protein is 75.08 kDa, and its p*I* value is 5.8. Leucine is the most abundant amino acid in the deduced peroxidase protein, comprising 10.3% of the amino acid residues. Other amino acids are present at a lower abundance, which ranges from 1.5 to 7.0%. The honey bee peroxidase protein shows 80% and 79% similarity with the peroxidases of *A. aegypti* and *C. quinquefasciatus*, respectively (ClustalW score) (Figure 2B).

File S5 summarizes the main characteristics of *AmeTwdl1*, *AmelTwdl2* and *Ampxd* CDSs and their deduced amino acid sequences.

### The *tweedle* and *peroxidase* genes are regulated during the molt cycle

**Figure 3:**
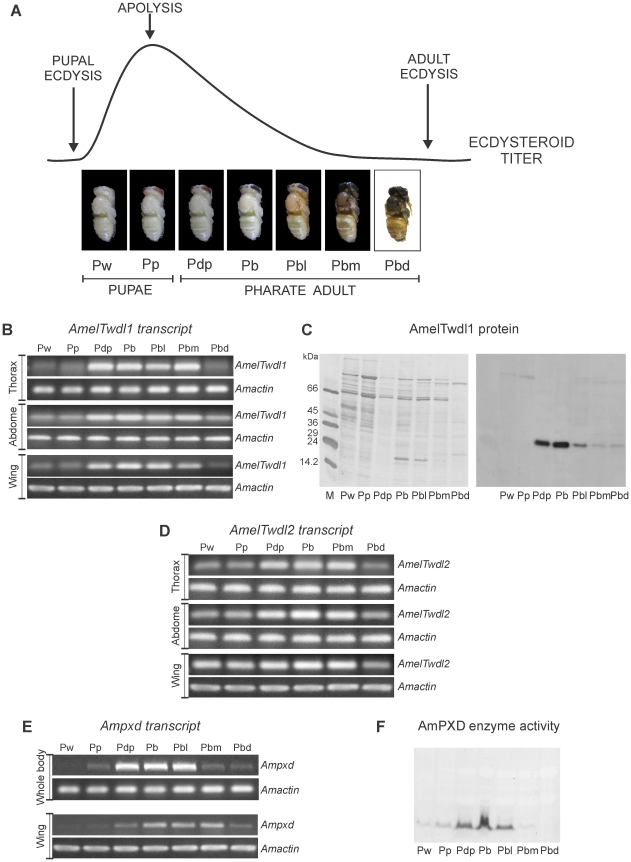
Expression of *AmelTwdl1*, *AmelTwdl2* and *Ampxd* genes during the pupal-to-adult development coordinated by ecdysteroid titer. (A) Hemolymph ecdysteroid titer redrawn from Pinto *et al*. (see ref. [59]), and the successive developmental phases in the interval between the pupal and adult ecdyses: Pw and Pp are early and late pupae; Pdp, Pb, Pbl, Pbm and Pbd are the successive pharate adult phases. (B) Transcriptional profile of *AmelTwdl1* in the thoracic, abdominal and wing integument. (C) Developmental profile of thoracic integument proteins stained with Coomassie Brillant Blue (at the left) and the AmeTwdl1 protein (at the right) as detected using Western blot and an antibody against a cuticle protein from *B. mori* (BmGRP2). (D) Transcriptional profile of *AmelTwdl2* in the thoracic, abdominal and wing integument. (E) Transcriptional profile of *Ampxd* in whole body extracts and wings. (F) AmPXD enzyme activity detected in thoracic integument samples using electrophoresis in polyacrylamide gels stained with a specific substrate. Transcript abundance was investigated by semiquantitative RT-PCR followed by electrophoresis of the amplified cDNAs in ethidium bromide-stained agarose gels. An *A. mellifera actin* gene, *Amactin*, was used as endogenous control.

The expression of *AmelTwdl1*, *AmelTwdl2* and *Ampxd* was studied in the thoracic, abdominal and wing integuments during the pupal-to-adult molt cycle coordinated by changes in ecdysteroid titer. Approximately one day after the pupal ecdysis, the ecdysteroid titer rises and reaches a peak, thus inducing apolysis of the pupal cuticle. The ecdysteroid titer then decreases gradually. The adult ecdysis occurs when the hormone reaches a basal level. Figure 3A shows ecdysteroid titer modulation and the sequential changes in the honey bee cuticle during pupal and pharate adult development.

### *AmelTwdl1* expression

The thoracic integument of pupae (Pw phase) and of the succeeding developmental phase where the pupal-to-adult apolysis takes place (Pp phase) exhibit very low levels of *AmelTwedl1* transcripts, but subsequently the abundance of this mRNA rapidly increases. High transcript levels are then maintained during almost all of the pharate adult stage, which comprises the time interval between the Pdp and Pbm phases, and this is followed by a decrease in transcript levels just before the ecdysis to the adult stage, at the Pbd phase (Figure 3B, upper panel). A similar pattern of expression occurs in the integument of the abdomen and wings (Figure 3B, middle and lower panels).

Western blots of honey bee proteins extracted from the thoracic integument and then probed with an antibody against the CP from *B. mori*, BmGRP2, revealed a band that matches the molecular mass of the deduced AmelTwdl1 protein (30.81 kDa), indicating that the antibody reacted with the product of the *AmelTwdl1* gene (observe in Figure 1A that the light-gray marked amino acid region of the BmGRP2 sequence used for antibody production almost completely matches a region in the AmelTwdl1 protein). In addition, and reinforcing this argument, we observed that the temporal pattern in the Western blots is similar to the *AmelTwdl1* transcriptional pattern. The AmelTwdl1 protein appears in the thoracic integument soon after apolysis in the Pdp phase, where it is abundant, and it remains at a high level in the next Pb phase, but then decreases gradually in the subsequent pharate adult phases (Pbl, Pbm and Pbd) (Figure 3C). A similar AmelTwdl1 expression profile was detected in the abdominal integument of the same developmental phases (data not shown).

### *AmelTwdl2* expression

The expression pattern of *AmelTwdl2* during the pupal-to-adult molt is similar to that observed for *AmelTwdl1*, although the differences in transcript levels among the developmental phases as detected by RT-PCR are not as striking. The level of *AmelTwdl2* transcripts in the thoracic integument is low during pupal development (Pw and Pp phases), increases in the earlier phase of the pharate adult development (Pdp) and remains high up to the penultimate pharate adult phase (Pbm) but then decreases in the last pharate adult phase (Pbd) (Figure 3D, upper panel). A similar pattern was observed in the abdominal and wing integuments (Figure 3D, middle and lower panels).

### *Ampxd* expression

The expression of *Ampxd* increases in the whole body during the early pharate adult phase (Pdp) and is maintained at a high level during the subsequent Pb and Pbl pharate adult phases and then the expression decreases in the final pharate adult phases (Pbm and Pbd) (Figure 3E, upper panel). A similar temporal expression pattern was observed using the wings as the RNA source, but with a slight delay in the onset of high expression to the Pb phase (Figure 3E, intermediate panel). The activity profile of the potential AmPXD enzyme was also tested using a specific substrate. The peroxidase activity (Figure 3F) matched the pattern of the *Ampxd* transcript (Figure 3E, upper panel), suggesting that the enzyme is the product of *Ampxd.*

### Ecdysteroid titers *versus* expression profiles of the *tweedle* and *peroxidase* genes

By contrasting the gene expression profiles (Figure 3B–F) with the changing hemolymph ecdysteroid titer that coordinates the pupal-to-adult molt (Figure 3A), we observed that the transition from low to high levels of transcripts and proteins occurred as the ecdysteroid titer was decreasing from the peak that triggers apolysis (in the Pp phase). This comparative analysis suggests that the expression of the genes *AmelTwdl1*, *AmelTwdl2* and *Ampxd* is activated by this pulse of ecdysteroids for the subsequent formation of the adult cuticle, which occurs as the hormone titer decreases during the pharate adult development. The decreased expression in late pharate adults (Pbd), which are ready to ecdyse to the adult stage, coincides with the advanced process of cuticle differentiation, as seen by its intense pigmentation and sclerotization.

**Figure 4:**
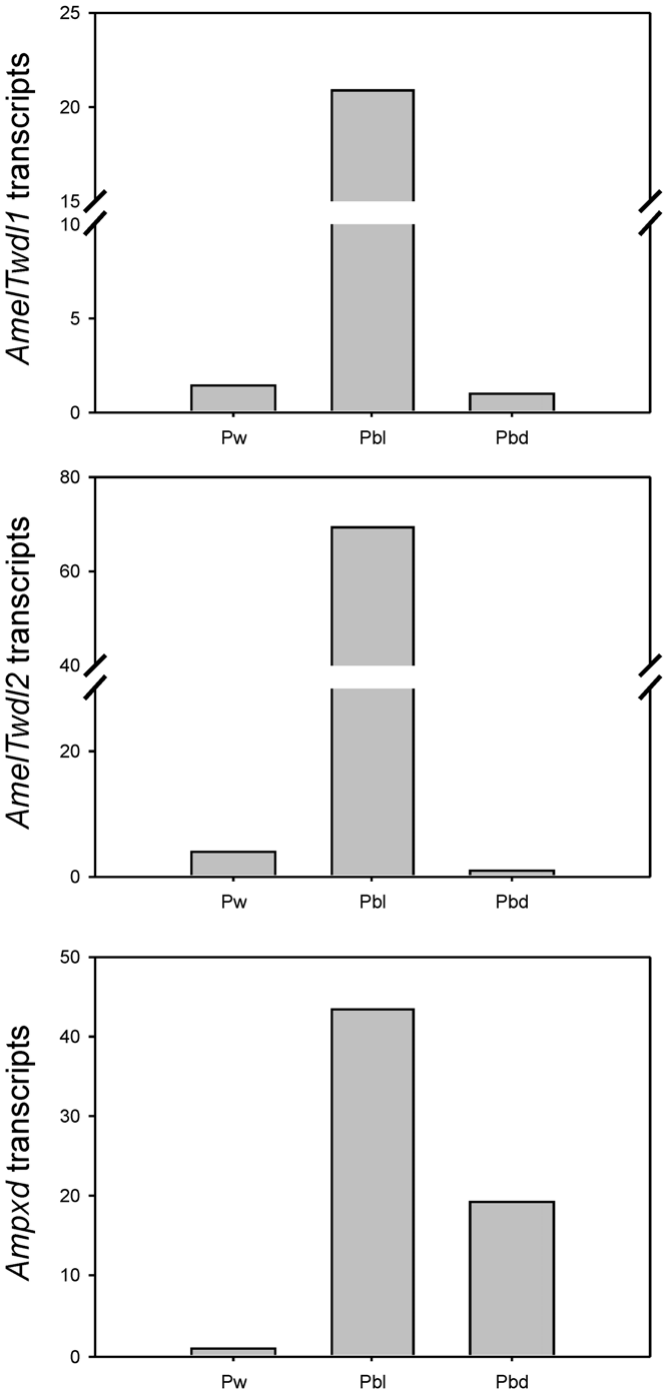
Relative quantification of *AmelTwdl1*, *AmelTwdl2* and *Ampxd* transcripts in the thoracic integument of pupae (Pw phase) and pharate adults (Pbl and Pbd phases). The *Amrp49* gene was used as endogenous control in real-time RT-PCR assays, and the relative amount of transcripts is given by 2^-ΔΔC^_T_. Each column represents the mean of two independent integument samples.

The transcriptional profiles shown in Figure 3 were corroborated by the results obtained by real time RT-PCR analysis using RNA samples from thoracic integuments. Figure 4 shows very low levels of the three transcript types in the pupal (Pw) thoracic integument, but the expression is significantly higher in the middle of the pharate adult development (Pbl phase), and subsequently decreases at the end of this period (Pbd phase). The levels of *AmelTwdl1*, *AmelTwdl2* and *Ampxd* transcripts were 15-, 18.6- and 45.5-fold higher, respectively, in the thoracic integument from Pbl pharate adults than in the integument from Pw pupae.

### Expression in different tissues

**Figure 5:**
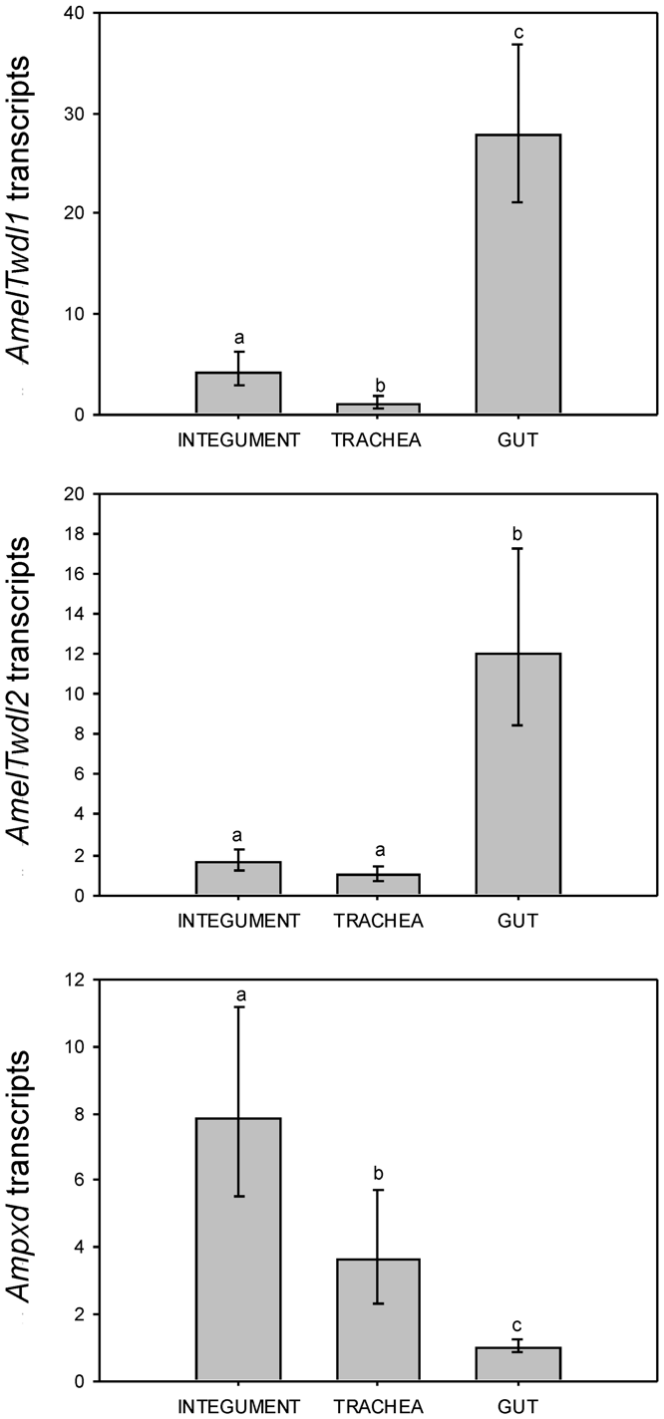
Relative quantification of *AmelTwdl1*, *AmelTwd2* and *Ampxd* transcripts in different tissues of the Pbl pharate adult. The *Amrp49* gene was used as endogenous control in real-time RT-PCR assays. The relative amount of transcripts is given by 2^-ΔΔC^_T_. Columns and bars represent means±SE of three independent samples prepared with each tissue. *AmelTwdl1* transcript levels were significantly different among the tested tissues, with a higher expression in the gut (p = 0.002). Similarly, *AmelTwdl2* showed a significantly higher expression in the gut than in integument and trachea (p = 0.010). In contrast, *Ampxd* showed a higher expression in integument than in the other tissues (p = 0.001). Statistical analysis was carried out with Jandel SigmaStat 3.1 software (Jandel Corporation, San Rafael, CA, USA). One Way Anova; post-hoc comparisons using Holm Sidak test p<0.05. Different letters above columns indicate statistical difference.

Real-time RT-PCR was used to quantify the levels of *AmelTwdl1*, *AmelTwdl2* and *Ampxd* transcripts in the gut and trachea, and the expression in these tissues was compared to the expression in the thoracic integument. The levels of *AmelTwd1* and *AmelTwdl2* transcripts were significantly higher in the gut than in the thoracic integument (6.6- and 7.2-fold, respectively) and trachea (27.8- and 12.1-fold, respectively). In contrast, *Ampxd* showed the higher expression in the thoracic integument, with 7.8- and 2.2-fold higher transcript levels than in the gut and trachea, respectively (Figure 5).

### Down regulation of the *tweedle* and *peroxidase* genes in ligated abdomens

**Figure 6:**
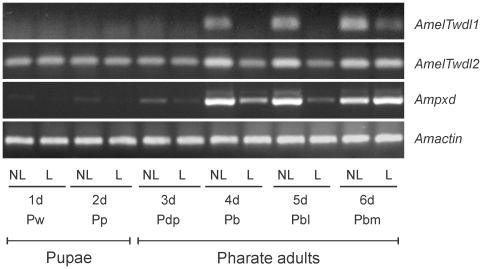
Abundance of *AmelTwdl1*, *AmelTwdl2* and *Ampxd* transcripts in the integument of ligated (L) abdomens compared to non-ligated controls (NL). Transcript levels were investigated at days (d) 1, 2, 3, 4, 5 and 6 after the abdominal ligature of newly ecdysed pupae. Transcript levels were assessed by semiquantitative RT-PCR assays followed by electrophoresis of the amplified cDNAs in ethidium bromide stained agarose gels. The *Amactin* gene was used as endogenous control.

The amounts of the *AmelTwdl1*, *AmelTwdl2* and *Ampxd* transcripts did not increase in the integument of ligated abdomens as they did in intact abdomens, at least up to the fifth day after ligation (Figure 6). By impairing the flow of hemolymph, and consequently of ecdysteroids, to the abdomen, the ligature also temporarily impaired the increase in the abdominal levels of *AmelTwdl1*, *AmelTwdl2* and *Ampxd* transcripts. This result suggests that abdominal exposure to the ecdysteroid peak that induces apolysis (see Figure 3) is critical for the increase in the levels of all of these transcripts in pharate adults. This result also reinforces the fact that *Ampxd* encodes a genuine cuticular peroxidase involved in cuticle sclerotization, which, like the CP genes studied here, depends on the ecdysteroid pulse for maximal activity.

However, the suppression of gene expression in the integument of ligated abdomens was transient, with a clear increase in transcript levels observed on the sixth day after ligation. This intriguing result could be explained by the hypothetical existence of a secondary source of ecdysteroids in the abdomen that would induce the expression of these genes on the sixth day (see the last item of the Discussion section).

## Discussion

### Structural features and expression patterns of *AmelTwdl1* and *AmelTwdl2* are consistent with roles in the formation of exoskeleton and other cuticles

The study of a mutation that affects *D. melanogaster* body shape led Guan *et al*. [49] to identify a new class of CPs, named Tweedle. Using one of these proteins (TweedleD) as a query, these authors identified Tweedle sequences in different insect genomes, including the honey bee genome. The alignment of these sequences has highlighted the four conserved blocks of amino acids that now define the Tweedle CP family (see Fig. 4 in [49]).

A consensus seems to exist among experts in CPs that the presence of the four conserved blocks of amino acids is sufficient for inclusion in the Tweedle class, despite richness in glycine residues. In fact, Cornman and Willis [50] included the 35% glycine-enriched AmelTwdl1 (then identified under the GenBank accession number gi:217330652) in a neighbor-joining phylogenetic analysis based on predicted Tweedle proteins of eight insect species. Futahashi *et al*. [51] also gave preference to the Tweedle motifs over glycine-rich characteristics and renamed the 36% glycine-enriched BmGRP2 (B. mori Glycine Rich Protein 2) as BmorCPT3, i.e., *B. mori Cuticle Protein Tweedle 3*. Furthermore, six *D. melanogaster* CPs containing both glycine-rich domains and the four amino acid blocks were included in the Tweedle class together with other twenty-one glycine-poor Tweedle proteins [49].

The other honey bee sequence containing the conserved Tweedle blocks was here named AmelTwdl2 (this sequence is also included in the above-mentioned phylogenetic analysis by Cornman and Willis [50] under the GenBank accession number gi:110766924). The only two *tweedle* genes in the honey bee genome differ considerably in structure and length. In contrast to *AmelTwdl1*, *AmelTwdl2* does not encode a glycine-rich protein, but a protein that has many occurrences of proline, with several PP repeats. Proline richness is a characteristic of CPs classified as CPLCP (Cuticular Protein of Low Complexity, Proline-rich) [50], where proline residues occur mainly as PY and PV. AmelTwdl2 only has one PY motif and does not have any PV motif, and therefore, by these criteria it should not be included in the CPLCP class. It is known that the amino acid composition of CPs influences the mechanical properties of cuticle, thus implying that AmeTwdl1 and AmelTwdl2 may somehow differ in their roles, but this is far from being completely understood.

Consistent with secretion by the epidermis for integration in the cuticle, N-terminal signal peptides were identified in the deduced AmelTwdl1 and AmelTwdl2 protein sequences. For AmelTwdl1 this was confirmed by sequencing of the gene.

*AmelTwdl1* and *AmelTwdl2* are expressed in the superficial integument (epidermis) covering the thorax, abdomen and wings. Both genes are up-regulated during the pharate adult development, period when synthesis and deposition of the adult cuticle take place, suggesting roles in cuticle renewal during the pupal-to-adult molt. By showing a close correspondence between *AmelTwdl1* transcript and protein profiles our data reinforce the assumption of a role in cuticle renewal. The modulated expression of the honey bee *tweedle* genes in the integument, and the findings that *tweedle* genes from *Drosophila* [49], and *B. mori* [51] are expressed in epidermis are consistent with a function in exoskeleton structuring.

We also verified that the expression of both *tweedle* genes in the honey bee integument is not exclusively linked to the formation of the adult cuticle, as these genes are similarly induced after apolysis of the larval cuticle, during the synthesis of the pupal cuticle (data not shown). The periodic expression of *AmelTwdl1* and *AmelTwdl2* in integument strongly suggests that their products are essential for exoskeleton formation at each molt cycle. This observation agrees with Cornman and Willis [50] data on expression levels of various *tweedle* genes during larval and pupal development of *A. gambiae*. Cornman and Willis [50] data are, in our understanding, consistent with the regulation of *tweedle* genes by ecdysteroid titer fluctuation during the succession of the molting events.

Therefore, like the majority of the CPR genes [52], the *tweedle* genes are important for exoskeleton reconstruction in the different developmental stages.

In addition of being active in epidermis, the honey bee *tweedle* genes are also expressed in other cuticle producing tissues, such as the trachea, the inner surface of which is lined by cuticle, and unexpectedly, the highest expression was detected in the gut. At least two of the *tweedle* genes of *Drosophila* (*TwdlC* and *TwdlE*) are also expressed in the foregut of late-stage embryos [49], thus evidencing a role for the Tweedle family proteins in the formation of the chitinous peritrophic membrane that lines the inner surface of the gut epithelium.

Proteins of the Tweedle family lack the R&R Consensus sequence that confers chitin-binding and cross-linking properties to the most abundant class of CPs, the CPR proteins [53]. Based on the prediction that the four blocks of amino acids in the Tweedle proteins form β-strands containing conserved aromatic residues that bind chitin, Tweedle proteins had been proposed to interact with chitin [49]. Recently, Tang *et al.* [54] confirmed this prediction by demonstrating that a Tweedle protein from *B. mori* (BmorCPT1) effectively binds chitin. Therefore, AmelTwdl1 and AmelTwdl2 must be part of the proteinaceous matrix that interacts with chitin to form the cuticle lining epidermis and trachea, and the gut peritrophic membrane.

### *Ampxd* encodes a peroxidase with a potential role in pharate-adult cuticle sclerotization

The oxidation of catechols to quinones for cuticle sclerotization may occur via the action of peroxidases and other enzymes [16]. Peroxidases, which are heme-containing enzymes that catalyze oxidative reactions, have been suggested to play a role in sclerotization based on the detection of specific activity in the cuticle through histochemical methods [13], [55], [56].

Cuticular peroxidases are believed to be secreted into the cuticle by means of an amino-terminal hydrophobic region that is cleaved by a peptidase, thus allowing exportation through the secretory pathway. However, using computer-based programs we could only find weak evidence of a candidate signal peptide in AmPXD. Similarly, no signal peptide has been recognized in the peroxidase of *A. aegypti*, although a signal peptide is clearly present in the *C. quinquefasciatus* peroxidase. Exceptions to the well-known mode of protein secretion dependent on a cleavable signal peptide have been reported in the literature. Some eukaryotic proteins can be efficiently secreted even if they lack a typical signal peptide [57], [58]. Further studies are needed to establish the mechanisms by which AmPXD and the peroxidase of *A. aegypti* reach the cuticle.

Transcripts of *Ampxd* were undetectable in the integument of larvae, pupae and ecdysed adult bees (data not shown). In contrast, *Ampxd* transcripts were found in significant amounts in the sclerotizing thoracic and wing integuments and in the trachea of pharate adult bees. These temporal and spatial patterns of *Ampxd* expression strongly suggest that it plays a role in sclerotization of the definitive exoskeleton and of the cuticle lining the lumen of the adult tracheal tree.

The temporal pattern of peroxidase activity during the pupal-to-adult development of the honey bee exactly matches the expression profile of the transcript. Thus, the detected enzyme activity was tentatively assigned to the *Ampxd* gene product, but this finding requires further confirmation.

### Ecdysteroid-dependent expression of *AmelTwdl1*, *AmelTwdl2* and *Ampxd* genes

An elevation in ecdysteroid titer in hemolymph of the honey bee pupae (Pp phase) [59] induces the detachment of the pupal cuticle and this is followed by the gradual synthesis and differentiation of the adult cuticle, which occur as the hormone titer declines [60]. Gene regulation by ecdysteroids in the context of cuticle renewal during molting and metamorphosis has been mainly described for *D. melanogaster* and *M. sexta* [19], [61]. The activity of several CP genes requires a pulse of ecdysteroids, i.e., a rapid increase followed by a decline to basal levels. Although this mode of gene regulation does not adapt to all CP genes, it has been clearly shown not only in *Drosophila* and *M. sexta*, but also in *B. mori*, *Tenebrio molitor* [62] and for the *AmelCPR14* gene in the honey bee [36]. Most likely, these genes are effectors, or late-response genes, that indirectly respond to ecdysteroids. The genes here studied follow the same mode of regulation by ecdysteroids, as suggested by the respective expression patterns and ligature experiments. The three genes showed increased expression after the ecdysteroid peak, when the hormone titer was declining. By preventing the increase in ecdysteroid concentration in abdomen, the ligature also inhibited the natural increase of *AmelTwdl1*, *AmelTwdl2* and *Ampxd* transcript levels in the abdominal integument, thus indicating that the ecdysteroid peak is critical for induction of these genes. The complete and correct formation of the adult cuticle was also prevented in the ligated abdomens, which remained non-pigmented and much less sclerotized, whereas the cuticle covering the head and thorax developed normally (see Fig. 5A in [11]). However, on the sixth day after ligature, we observed a consistent increase in the levels of the three types of transcripts in the abdominal integument. This result may be explained by assuming the existence of a secondary ecdysteroid source in the abdomen in addition to the primary source, i.e., the ecdysteroid-producing prothoracic glands. A later increase in ecdysteroid titer in ligated abdomens of *A. mellifera* was demonstrated using a radioimmunoassay [11]. Abdominal cells, most likely epidermal cells and oenocytes, were inferred to be the secondary sources of ecdysteroids. Several authors have referred to active secondary ecdysteroid sources during the late steps of insect metamorphosis when the prothoracic glands have completely or nearly completely disappeared due to programmed cell death. The existence of secondary ecdysteroid sources was also hypothesized in experiments using isolated insect body parts or explanted tissues incubated *in vitro* ([18], [63] and references therein). Ecdysteroids were also detected in *in vitro* incubations of fat body-adhered to cuticle, and other tissues of the adult worker honey bee [64]. We suggest that in ligated abdomens of honey bees, a late and transient release of abdominal ecdysteroids caused the increase in the levels of *AmelTwdl1*, *AmelTwdl2* and *Ampxd* transcripts at the sixth day after ligation.

In conclusion, by validating the predicted *tweedle* sequences and by revealing a novel gene, *Ampxd*, we contributed to expand the set of honey bee genes (*AmelCPR14* [36], *Amlac2* [11] and the three apidermin genes, *apd-1*, *apd-2* and *apd-3* [37]) that are experimentally confirmed as having a role in cuticle formation. In addition, *AmelTwdl1*, *AmelTwdl2* and *AmPxd* were herein characterized in terms of structure, temporal- and tissue-specific expression, and regulation by ecdysteroids. Taken together, our data highlighted the roles of these genes in the context of the ecdysteroid-coordinated pupal-to-adult molt cycle.

## Supporting Information

File S1 **Specific primer sequences used for sqRT-PCR, qRT-PCR, and for sequencing the** ***AmelTwdl*** **and** ***Ampxd*** **genes. The GenBank accession number of each gene is underlined.**
(DOC)

File S2 ***AmelTwdl1*** **nucleotide sequence and translated product.** Only the last nucleotides (underlined) were not validated by sequencing the cDNA. Stop codon is in red. Part of the 5′UTR (blue letters) was also confirmed by sequencing the cDNA. Signal peptide is marked with a dashed line. The sequenced cDNA was deposited in the GenBank under the accession number FJ380949.1 (ACJ38118.1 for its conceptual translation product).
(DOC)

File S3 ***AmelTwdl2*** **nucleotide sequence and translated product.** Nucleotides encoding the N-terminal region (underlined) were not validated by sequencing the cDNA. Stop codon is in red. Part of the 3′UTR (blue letters) was also confirmed by sequencing the cDNA. The deduced signal peptide is marked with a dashed line. The sequenced cDNA was deposited in the GenBank under the accession number HM481255.2 (ADK73965.2 for its conceptual translation product).
(DOC)

File S4 ***Ampxd*** **nucleotide sequence and translated product.** The region not validated by sequencing the gene is underlined. Stop codon is in red. Signal peptide is marked with a dashed line. The partially sequenced CDS was deposited in the GenBank under the accession number GU785071.2 (ADE45321.2 for its conceptual translation product).
(DOC)

File S5 **Characteristics of** ***AmelTwdl1*** **,** ***AmelTwdl2*** **and** ***Ampxd*** **CDSs and their respective predicted proteins.**
(DOC)
