# Supplementary material for: Ecdysteroid-Dependent Expression of the Tweedle and Peroxidase Genes during Adult Cuticle Formation in the Honey Bee, Apis mellifera
Source: PLoS One. 2011 May 31;6(5):e20513. doi: 10.1371/journal.pone.0020513 (PMC3105072; doi:10.1371/journal.pone.0020513)
Supplement: File S1 — Specific primer sequences used for sqRT-PCR, qRT-PCR, and for sequencing the AmelTwdl and Ampxd genes. The GenBank accession number of each gene is underlined. (DOC) [file pone.0020513.s001.doc]

**File S1. Specific primer sequences used for sqRT-PCR, qRT-PCR, and for sequencing the *AmelTwdl* and *Ampxd* genes. The GenBank accession number of each gene is underlined.**

| **Genes** | **Primers** |
| --- | --- |
| *AmelTwdl1*  FJ380949.1 | Sq and qRT-PCR  ***f*** - 5’CAAATCCAAGGAACAGCAGC3’  ***r*** - 5’GTGCTCCATAAGAGGAGCTG3’ |
|  | Sequencing  ***f* 1** - 5’CAGACGGTCTCATCAGCATA3’  ***r*1 -** 5’GTGCTCCATAAGAGGAGCTG3’  ***f* 2** - 5’GAAGCCGGTTACTCTTACTC3’  ***r*2** - 5’TGTCGCGGAGCATAGACATA3’ |
| *AmelTwdl2*  HM481255.2 | Sq and qRT-PCR  ***f*** - 5’GTGGTTCTTCCCACTCAAGC 3’  ***r*** - 5’TATCCGAAGGAGGTTGTGCT 3’ |
|  | Sequencing  ***f* 1** - 5’CGAAATCGAAAGCGAGTACAA3’  ***r*1 –** 5’GTTTGCTAGGTTGCGTTGGT3’  ***f* 2** - 5’CAGAGGCTCCGGAATACAAA3’  ***r*2** - 5’CGCGTGTTTCGAGTATTTGTT3’ |
| *Ampxd*  GU785071.2 | qRT-PCR  ***f*** - 5’AGAGTGAACGAGCAGCTAGT3’  ***r*** - 5’ATACAACGTTTCATCGTCCCA3’ |
|  | sqRT-PCR  ***f*** -5’GCTGTATCAAGAAGCAAGAAG3’  ***r*** -5’CAAATTCACGTCCTCGTTGC3’ |
|  | Sequencing  ***f* 1** - 5’ggatatcagtgcgaaccttg3’  ***r*1 –** 5’cgtgataaccctcgtcaggA3’  ***f* 2** - 5’tcctgacgagggttatcacg3’  ***r*2** - 5’gccgatggcaacattcctcc3’  ***f* 3** - 5’ggaggaatgttgccatcggc3’  ***r*3** - 5’gatggattcacgctttcgtc3’  ***f* 4** - 5’gctgtatcaagaagcaagaag3’  ***r*4** - 5’caaattcacgtcctcgttgc3’ |
| *Amrp49*  AF441189.1 | qRT-PCR  ***f*** - 5’CGTCATATGTTGCCAACTGGT3’  ***r*** - 5’TTGAGCACGTTCAACAATGG3’ |
| *Amactin*  AB023025.1 | sqRT-PCR  ***f*** - 5’TGCCAACACTGTCCTTTCTG3’  ***r*** - 5’AGAATTGACCCACCAATCCA3’ |
