# Supplementary material for: Ecdysteroid-Dependent Expression of the Tweedle and Peroxidase Genes during Adult Cuticle Formation in the Honey Bee, Apis mellifera
Source: PLoS One. 2011 May 31;6(5):e20513. doi: 10.1371/journal.pone.0020513 (PMC3105072; doi:10.1371/journal.pone.0020513)
Supplement: File S2 — AmelTwdl1 nucleotide sequence and translated product. Only the last nucleotides (underlined) were not validated by sequencing the cDNA. Stop codon is in red. Part of the 5′UTR (blue letters) was also confirmed by sequencing the cDNA. Signal peptide is marked with a dashed line. The sequenced cDNA was deposited in the GenBank under the accession number FJ380949.1 (ACJ38118.1 for its conceptual translation product). (DOC) [file pone.0020513.s002.doc]

**File S2. *AmelTwdl1* nucleotide sequence and translated product.**

Only the last nucleotides (underlined) were not validated by sequencing the cDNA. Stop codon is in red. Part of the 5’UTR (blue letters) was also confirmed by sequencing the cDNA. Signal peptide is marked with a dashed line. The sequenced cDNA was deposited in the GenBank under the accession number FJ380949.1 (ACJ38118.1 for its conceptual translation product).

1 - **CAGACGGTCTCATCAGCATAGGTG**ATGGGTCGAGATGCGAGTGGGGCCAGAGCTAGTATA - 60

1 - M G R D A S G A R A S I - 12

61 - AAAGAGGGTCCGTGGCATCCGTGGAGCATCAGTCCTCAAATCCAAGGAACAGCAGCTACC - 120

13 - K E G P W H P W S I S P Q I Q G T A A T - 32

121 - ATGAGGGCATTCATGATCGTGGTGCTCGCCGCTTCAGCAATGGCGCGGCCAGAAGCCGGT - 180

33 - M R A F M I V V L A A S A M A R P E A G - 52

181 - TACTCTTACTCTCAACCCAGCTCCTCTTATGGAGCACCAGGAGGTGGAACTACCGGAATT - 240

53 - Y S Y S Q P S S S Y G A P G G G T T G I - 72

241 - GGTGGAGGATTGGGTGGTGGATTAGGTGGTGGACTTGGAGGTGGACATGGCGGTGGAATC - 300

73 - G G G L G G G L G G G L G G G H G G G I - 92

301 - GGCGGTGGATTGGGTAGTGGATTAGGCGGTGGACTTGGCAGTGGACTCGGTGGTGGATTC - 360

93 - G G G L G S G L G G G L G S G L G G G F - 112

361 - GGTGGAGGTTTCGGTGGTGGCTTTGGAGGGGGCATCGGTGGCGGTGGCGGTGGTGGTTTT - 420

113 - G G G F G G G F G G G I G G G G G G G F - 132

421 - GGTGGAGGCGTTGGAGGTGGCTTCGGAGGTGGTTCTCTGATTCAAAAACACATTTACGTG - 480

133 - G G G V G G G F G G G S L I Q K H I Y V - 152

481 - CATGTGCCACCACCAGAAGCTCCAGAAGACAGACCAGCTAGACCTATTGCACCACCACCA - 540

153 - H V P P P E A P E D R P A R P I A P P P - 172

541 - CCACCTCAGAAACATTACAAGATTATATTCATCAAGGCGCCTACACCACCAACTCCCACT - 600

173 - P P Q K H Y K I I F I K A P T P P T P T - 192

601 - GCTCCTGTCATTCCTGCTCTTCCTCAACAAGATGAACAGAAAACATTGATTTACGTGTTG - 660

193 - A P V I P A L P Q Q D E Q K T L I Y V L - 212

661 - GTTAAAAAGCCAGAAGAAGCGCCAGAAATCACTTTGCCCACCATTGCTCCTACCCAACCG - 720

213 - V K K P E E A P E I T L P T I A P T Q P - 232

721 - AGCAAACCAGAAGTATACTTCATCAAATATAAGACTCAGAAGGAAGTTACCTCTGGTGGT - 780

233 - S K P E V Y F I K Y K T Q K E V T S G G - 252

781 - GGAGGTGGTGGCGGCGGTGGTGGAATCGGCGGTGGAATCGGCGGTGGAATTGGAGGCGGA - 840

253 - G G G G G G G G I G G G I G G G I G G G - 272

841 - ATTGGCGGAGGAATTGGTGGCGGAATCGGCGGAGGAATCGGTGGAGGAATCGGTGGAGGA - 900

273 - I G G G I G G G I G G G I G G G I G G G - 292

901 - ATCGGCGGAGGAATCGGTGGTGGATCCGGTGGAGGCATCGGAGGACTGGACGGTCATGGC - 960

293 - I G G G I G G G S G G G I G G L D G H G - 312

961 - GGCAGCGGACCAAGCGGACCCAGCACATCTTACGGGACACCCGGCGCTTCAGGGCC**TTAC**TAG - 1023

313 - G S G P S G P S T S Y G T P G A S G P Y * - 332
