# Supplementary material for: Ecdysteroid-Dependent Expression of the Tweedle and Peroxidase Genes during Adult Cuticle Formation in the Honey Bee, Apis mellifera
Source: PLoS One. 2011 May 31;6(5):e20513. doi: 10.1371/journal.pone.0020513 (PMC3105072; doi:10.1371/journal.pone.0020513)
Supplement: File S3 — AmelTwdl2 nucleotide sequence and translated product. Nucleotides encoding the N-terminal region (underlined) were not validated by sequencing the cDNA. Stop codon is in red. Part of the 3′UTR (blue letters) was also confirmed by sequencing the cDNA. The deduced signal peptide is marked with a dashed line. The sequenced cDNA was deposited in the GenBank under the accession number HM481255.2 (ADK73965.2 for its conceptual translation product). (DOC) [file pone.0020513.s003.doc]

**File S3. *AmelTwdl2* nucleotide sequence and translated product.**

Nucleotides encoding the N-terminal region (underlined) were not validated by sequencing the cDNA. Stop codon is in red. Part of the 3’UTR (blue letters) was also confirmed by sequencing the cDNA. The deduced signal peptide is marked with a dashed line. The sequenced cDNA was deposited in the GenBank under the accession number HM481255.2 (ADK73965.2 for its conceptual translation product).

1 - ATGTTAATGTCTTTCCTGTGTTCTCCTCAAAATACCACGCTACTATTGGTAGCTTGTACA - 60

1 - M L M S F L C S P Q N T T L L L V A C T - 20

61 - ACGAGGGTATATTCGAAACCAGCACCGGAACCACCCAGCTCTTACTTCCCCCCATCGAAT - 120

21 - T R V Y S K P A P E P P S S Y F P P S N - 40

121 - GGCGGCAGTTTGTCATCCGGTAATTATGGTCCCCCGTCGCTTCCAGATCGGTATGGGCCG - 180

41 - G G S L S S G N Y G P P S L P D R Y G P - 60

181 - CCCCAGCAACAACCGATCGTCCACAAACACGTTTACGTCCACGTGCCACCTCCAGAGGCT - 240

61 - P Q Q Q P I V H K H V Y V H V P P P E A - 80

241 - CCGGAATACAAACCGCCGAAATACATACCACCCGCGGCACCACCGCAGAAACATTACAAG - 300

81 - P E Y K P P K Y I P P A A P P Q K H Y K - 100

301 - ATAGTGTTTATAAAGGCGCCAACGCCACCTACGCCAACAGCACCAGCTCTACCACCATTA - 360

101 - I V F I K A P T P P T P T A P A L P P L - 120

361 - CCGCCTCAAGACGAAGAAAAAACTTTGATCTACGTTCTGGTGAAAAAACCGGAGGAAGCG - 420

121 - P P Q D E E K T L I Y V L V K K P E E A - 140

421 - CCAGAAGTGGTTCTTCCCACTCAAGCACCAACGCAACCTAGCAAACCGGAAGTTTATTTC - 480

141 - P E V V L P T Q A P T Q P S K P E V Y F - 160

481 - ATTCGATACAAGACACAGAAAGAATCTCAAAATGTGGAATATGGACCTCCAGCACAACCT - 540

161 - I R Y K T Q K E S Q N V E Y G P P A Q P - 180

541 - CCTTCGGATAACTATGGAGCTCCACCATCAAGTCCTGGCGGACCTTATTAAATAATTTTA - 600

181 - P S D N Y G A P P S S P G G P Y * - 196

601 - TTACAAATGATCTATAAATCTCCTATGAAACAAATACTCGAAACACGCG - 649
