# Supplementary material for: Ecdysteroid-Dependent Expression of the Tweedle and Peroxidase Genes during Adult Cuticle Formation in the Honey Bee, Apis mellifera
Source: PLoS One. 2011 May 31;6(5):e20513. doi: 10.1371/journal.pone.0020513 (PMC3105072; doi:10.1371/journal.pone.0020513)
Supplement: File S4 — Ampxd nucleotide sequence and translated product. The region not validated by sequencing the gene is underlined. Stop codon is in red. Signal peptide is marked with a dashed line. The partially sequenced CDS was deposited in the GenBank under the accession number GU785071.2 (ADE45321.2 for its conceptual translation product). (DOC) [file pone.0020513.s004.doc]

**File S4. *Ampxd* nucleotide sequence and translated product.**

The region not validated by sequencing the gene is underlined. Stop codon is in red. Signal peptide is marked with a dashed line. The partially sequenced CDS was deposited in the GenBank under the accession number GU785071.2 (ADE45321.2 for its conceptual translation product).

1 - ATGTGGAGACCAGAGGATTTAGCCACGGTTGGAGAACTTTTATTGGATATCAGTGCGAAC - 60

1 - M W R P E D L A T V G E L L L D I S A N - 20

61 - CTTGTACAAACATACGGCCTAACCTTCGAAGAAATCGAAAAAAGTCTACCCTTAATCGAC - 120

21 - L V Q T Y G L T F E E I E K S L P L I D - 40

121 - ACTTCGAAGACCTTAATTCGCGAGGTGTGCCCTGCCTTTTTGAGCAACGTGGAATGTCGT - 180

41 - T S K T L I R E V C P A F L S N V E C R - 60

181 - GCTGGTAAATATAGAAGAAACGATGGCTTGTGCACAAATTTACAAAATCCGACATGGGGC - 240

61 - A G K Y R R N D G L C T N L Q N P T W G - 80

241 - GCCACATTAGCGCCCTTTCAAAGAGTGCTGAGTCCACGATTCGCGGACGGTCTAACAGCG - 300

81 - A T L A P F Q R V L S P R F A D G L T A - 100

301 - CCTAGAATATCGGTGACCAGTCATGATTTACCATTATCACGCATAGTGTCACGCACTATG - 360

101 - P R I S V T S H D L P L S R I V S R T M - 120

361 - CATCCTGACGAGGGTTATCACGATCATGCTGGTACAGTCATGGTCATCGCCTGGGGGCAG - 420

121 - H P D E G Y H D H A G T V M V I A W G Q - 140

421 - TTTATGGATCACGACTATACGCTAACTGGAACGCCTCTAGATCCTTTGAACCGAAACGAC - 480

141 - F M D H D Y T L T G T P L D P L N R N D - 160

481 - CCGGAGGAATGTTGCCATCGGCCACCGCACCTGAAGAATCCCTATTGCAACGAGATTCTT - 540

161 - P E E C C H R P P H L K N P Y C N E I L - 180

541 - ATACCGGAAGACGATTATTTCTACAGACTGTTCAACGTGAAATGCATGGACTTCGTTCGC - 600

181 - I P E D D Y F Y R L F N V K C M D F V R - 200

601 - GCCTTTCCTGCTGTACGACCTGGATGCCGACTCGGCTCTCGTGTGCCTTTCAATCTTCTT - 660

201 - A F P A V R P G C R L G S R V P F N L L - 220

661 - ACCGGTGTGCTCGATGGGAACACGGTGTATGGAATCACGGAATCATTCGCTAGGAAGCTA - 720

221 - T G V L D G N T V Y G I T E S F A R K L - 240

721 - CGGGCTGGTTATGGAGGATTGTTACGCATGAATCCAGTGTTCTCAGAATACGGACTGAAG - 780

241 - R A G Y G G L L R M N P V F S E Y G L K - 260

781 - GATTTATTACCGCTCAAGCTGGATATTCCGGACGAGGGATGCACCCGGCCGAATCGATCG - 840

261 - D L L P L K L D I P D E G C T R P N R S - 280

841 - ATGTACTGTTTCGAGGCGGGTGAGATAAGAGTGAACGAGCAGCTAGTGTTAACCTGCATG - 900

281 - M Y C F E A G E I R V N E Q L V L T C M - 300

901 - CATACTTTGATGGCACGTGAGCATAATCGAATTGCGAAGACGTTAATTCAAATAAATCCT - 960

301 - H T L M A R E H N R I A K T L I Q I N P - 320

961 - CATTGGGACGATGAAACGCTGTATCAAGAAGCAAGAAGAATCGTTATTGCTGAAATTCAA - 1020

321 - H W D D E T L Y Q E A R R I V I A E I Q - 340

1021 - CATATCACTTATAATGAATTTCTACCCATTTTACTTGGCAAAGATGTCATGGAAAAATTT - 1080

341 - H I T Y N E F L P I L L G K D V M E K F - 360

1081 - GGGCTCCTCCTTGAAAAAAATAGCTATTGGGATGGTTACGACGAAAGCGTGAATCCATCT - 1140

361 - G L L L E K N S Y W D G Y D E S V N P S - 380

1141 - GTGATCGATGCTTTCGCTTCTGCAGCCTTCAGATTCGGACACTCATTATTGCCAACGGCA - 1200

381 - V I D A F A S A A F R F G H S L L P T A - 400

1201 - GTAGAAAGGTGGAGTAAAGCTCATAAATTTATTGCTTCGAAAAGACTATCAGATTTGATT - 1260

401 - V E R W S K A H K F I A S K R L S D L I - 420

1261 - AGAAGACCGTTTGATTTGTATCGTGCTGGAGTTTTCGACGAATACATTATGGGGTTGATG - 1320

421 - R R P F D L Y R A G V F D E Y I M G L M - 440

1321 - AACCAAGTTGCTCAAGCTATGGACGATTCTATCACGCAAGAGGTGACAAATCATTTATTT - 1380

441 - N Q V A Q A M D D S I T Q E V T N H L F - 460

1381 - AAAAAAGTTGGAGCTAAGTTTGGATTGGATCTGGTCTCATTTAATATGCAACGAGGACGT - 1440

461 - K K V G A K F G L D L V S F N M Q R G R - 480

1441 - GAATTTGGTATTCCAAGTTATATGGAATTCAGAAAATTCTGTGGACTTCCTTGGGTAGAC - 1500

481 - E F G I P S Y M E F R K F C G L P W V D - 500

1501 - ACTTTTGACGAGCTTCACGGTTCCATGCCAAACGAAACGATCAGACGCTATAGCTCGATT - 1560

501 - T F D E L H G S M P N E T I R R Y S S I - 520

**File S4 (continued)**

1561 - TTTGAGCATCCAGCTGACGTCGATCTCTGGTCCGGTGGTGTATCCGAGAGACCACTTCCG - 1620

521 - F E H P A D V D L W S G G V S E R P L P - 540

1621 - GGTAGTATGCTCGGCCCAACCTTCGCCTGTATAATCGCCACGCAATTCAGTTATTCCCGT - 1680

541 - G S M L G P T F A C I I A T Q F S Y S R - 560

1681 - CGAGGCGACCGATTTTGGTACGAGTTGCCGAACCAGCCATCGTCCTTCACTCTCGATCAA - 1740

561 - R G D R F W Y E L P N Q P S S F T L D Q - 580

1741 - CTGAATGAGATCCGAAAAATAAAACTCGCTAGAGTGATCTGCGACAACACGGATTTGATC - 1800

581 - L N E I R K I K L A R V I C D N T D L I - 600

1801 - GATACTATACAGATTTATCCCATGGTATTACCCGATCATGAAATAAACCCACGAGTACCC - 1860

601 - D T I Q I Y P M V L P D H E I N P R V P - 620

1861 - TGTCGAAGCGGCGTCCTGCCCAGTATGGATCTAACCAAGTGGGCAGAATTCCCAACCGCG - 1920

621 - C R S G V L P S M D L T K W A E F P T A - 640

1921 - AATCATGCTCAATACGTGAGTAATATAGCGGAGACGTACCAGGCATATGGAAAATAA - 1977

641 - N H A Q Y V S N I A E T Y Q A Y G K * - 658
