# Supplementary material for: Ecdysteroid-Dependent Expression of the Tweedle and Peroxidase Genes during Adult Cuticle Formation in the Honey Bee, Apis mellifera
Source: PLoS One. 2011 May 31;6(5):e20513. doi: 10.1371/journal.pone.0020513 (PMC3105072; doi:10.1371/journal.pone.0020513)
Supplement: File S5 — Characteristics of AmelTwdl1 , AmelTwdl2 and Ampxd CDSs and their respective predicted proteins. (DOC) [file pone.0020513.s005.doc]

**File S5. Characteristics of *AmelTwdl1*, *AmelTwdl2* and *Ampxd* CDSs and their respective predicted proteins.**

| **Genes** | **Gene**  **Characteristics** | | | **Conceptual product characteristics** | | | | |
| --- | --- | --- | --- | --- | --- | --- | --- | --- |
|  | **Complete CDS**  **(nt)** | **CDS**  **Exons** | **Chromosomal**  **Region** | **Amino**  **acids** | **^1^Mol. mass**  **(kDa)** | **^1^pI** | **^2^Signal**  **peptide** | **Motifs** |
| *AmelTwdl1* | 999 | 3 | GroupUn.1241 | 332 | 30.81 | 9.2 | Yes | Conserved blocks  I, II, III and IV [49] |
| *AmelTwdl2* | 591 | 3 | GroupUn.592 | 196 | 21.36 | 8.4 | Yes |  |
| *Ampxd* | 1977 | 13 | GroupUn.1247 and Group4.3 | 658 | 75.08 | 5.8 | Yes | Animal haem peroxidase domain,  (pfam03098) |

^1^ Calculated using <http://expasy.org/tools/pi_tool.html>; ^2^Calculated using SignalP.
